# From Generative Molecular Design to Agentic Artificial Intelligence in Drug Discovery

**DOI:** 10.1002/cmdc.70564

**Published:** 2026-11-05

**Authors:** Andrea Mastropietro, Jürgen Bajorath

**Affiliations:** ^1^ Department of Life Science Informatics and Data Science B‐IT, LIMES Program Unit Chemical Biology and Medicinal Chemistry Rheinische Friedrich‐Wilhelms Universität Bonn Germany; ^2^ Lamarr Institute for Machine Learning and Artificial Intelligence Rheinische Friedrich‐Wilhelms‐Universität Bonn Bonn Germany; ^3^ Data Science Center Nara Institute of Science and Technology Ikoma Nara Japan

**Keywords:** AI agent systems, artificial intelligence, drug discovery, generative models, molecular design

## Abstract

Over the past decade, artificial intelligence (AI) has increasingly influenced the early stages of drug discovery, which focus primarily on target evaluation and the design or identification of new active small molecules or biologics. Deep neural networks adapted from areas such as natural language processing and computer vision have provided novel opportunities for generative modeling and human–machine interactions. State‐of‐the‐art AI models for generative molecular design have emerged, but remain only little understood so far. Furthermore, new computational systems are being devised rapidly that are expected to significantly transform the drug discovery landscape in the near future. These developments pose challenges to computer‐aided drug discovery in various ways. In the following, we discuss these opportunities and challenges, with a focus on molecular design.

## Graphical abstract

Generative artificial intelligence (AI) provides new opportunities for drug discovery. Moreover, emerging agentic AI systems are intended to become increasingly autonomous to automate research activities along the drug development pipeline. Principles from explainable and trustworthy AI must be considered to support the further development and wider adoption of such systems.

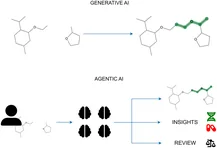

## 1 Preface

In the first part of this perspective, a concise overview of AI approaches in computer‐aided drug discovery is presented. This section aims to highlight the potential of AI methods for early‐phase drug discovery. Subsequently, challenges and open questions associated with these approaches are discussed. Finally, conclusions and a forward‐looking view of AI in drug discovery are presented. The discussion is supported with recent literature examples that are representative of current trends in this field. Most of the cited studies report on new AI‐centered methodologies and/or applications of such methods, while experimental validation of computational results remains relatively rare.

## 2 Deep Learning in Drug Discovery

Deep neural networks (DNNs) were adapted in computer‐aided drug discovery about a decade ago and initially applied for machine learning tasks such as compound design or molecular property prediction [1, 2, 3]. Areas of high relevance for drug discovery that were substantially impacted by DNNs included synthesis planning [4, 5], generative molecular design [6, 7], protein structure prediction [8, 9], and protein design [10, 11]. Protein language models (PLMs) [12, 13, 14] and chemical language models (CLMs) [15, 16] were adopted from natural language processing. These models operate based on textual representations of sequences or structures and rapidly gained popularity for generative design. For instance, PLMs were used to generate protein sequence embeddings [12, 13] to implicitly capture characteristic structural and functional information [14], which provided new opportunities for protein design [17]. Furthermore, CLMs significantly increased the ability to generate large volumes of highly diverse small molecules or new compounds conditioned on desired molecular properties [2, 3] and made it possible to address off‐the‐beaten‐path design tasks [16].

## 3 Generative Models

The first PLMs and CLMs were based on recurrent neural network architectures such as long short‐term memory (LSTM) cells [18] often used in encoder–decoder frameworks. These architectures were increasingly replaced by transformers with their hallmark attention mechanism (introduced in 2017) [17, 19, 20]. Transformers consist of encoder and/or decoder units with multiple self‐attention modules [19]. In these modules, several attention functions process the input sequence in parallel to generate long‐range dependencies between tokens. In addition, dependencies between input and output sequences are determined through cross‐attention.

Over the past 5 years, diffusion models, which were originally mostly applied in image generation, have also been increasingly used for small molecule and protein design [21]. Diffusion models learn input data distributions and, during the forward (diffusion) phase, add noise to the distributions that is then removed during the reverse (denoising) phase to reconstruct the input [21]. To this end, the popular denoising diffusion probabilistic model (DDPM) variant uses Markov chains to gradually generate a Gaussian data distribution during the forward phase, from which noisy data is sampled during the reverse phase until a noise‐free sample is obtained [22]. In molecular design, diffusion models use graph‐ or coordinate‐based input, and the generative process is often conditioned on embedded structural or property constraints (see below) [23].

In addition to task‐specific models, large language models (LLMs) are also used for molecular design [24, 25]. LLMs are based on the transformer architecture and learn statistical patterns in textual data representations to predict the next token in a sequence based on the preceding context and learned probability distributions. LLM operations generally require tokenization of textual representations, extensive training for transformer learning with attention mechanism, and prompt‐directed output generation. Multimodal LLMs enable the use of graph, numerical, property, or image data in addition to text [25, 26]. However, the interactive use of LLMs for scientific applications requires careful consideration (see below).

## 4 Structure‐Based Design

While the initial phase of deep generative modeling in drug discovery primarily focused on de novo small molecule design, in recent years, structure‐based drug design became a focal point of AI approaches in multiple ways. For instance, molecular docking, the most popular target structure‐based approach for computational hit identification, was combined with geometric deep learning for binding pose prediction [27]. In addition, docking was augmented with transformer modeling in different ways, for instance, by extracting chemical features from binding sites to aid in posing and scoring [28]. Furthermore, DDPMs have been used for docking to generate poses from randomized ligand conformations [29]. Moreover, diffusion models have been increasingly adopted for de novo structure‐based design by including principles from geometric deep learning. Importantly, equivariant diffusion models render molecule generation independent of transformations in three dimensions [30] and have been applied to generate new ligands de novo within the structural and property constraints of protein binding pockets [30] or through fragment‐based design [31]. In protein design, equivariant diffusion models have substantially advanced the generation of various protein–protein complexes, new enzymes, antibodies conditioned on specific antigens, or binding proteins conditioned on active small molecules or RNA [11]. Furthermore, the transformer architecture of AlphaFold2 for protein structure prediction was modified and extended with conditional DDPMs for the generation of protein–ligand complexes [32]. It is often not attempted to clearly determine the extent to which AI models improve the performance of conventional structure‐based methods. For example, new AI‐driven docking approaches and their pros and cons are often controversially viewed. However, a recent trend is the increasing use of diffusion models for protein design as well as the design of protein–ligand complexes. In several recent studies including experimental evaluation of new designs, promising results are reported.

## 5 Explainable AI (XAI)

Predictive AI models are black boxes [33, 34], which means that the inner workings to generate their output are not understandable for humans. Concepts from XAI [35] help to rationalize how these models arrive at their predictions. The field of XAI encompasses the development and application of methods to shed light on the inner workings of machine learning models, generate computational explanations of predictions, and uncover the associated data patterns [35, 36, 37]. In interdisciplinary research areas such as drug discovery, explanations of predictions that are interpretable in a chemically and/or biologically intuitive manner are of great importance for machine learning in order to impact experimental programs [38]. Among XAI approaches, feature attribution methods such as adaptations of the Shapley value concept are widely applied in the molecular sciences [39, 40]. Such feature attribution methods are model‐agnostic, that is, they are applicable to AI models of any architecture and complexity. However, the resulting computational explanations that quantify feature importance for predictions typically require additional analysis for human interpretation, such as feature mapping [40]. For XAI, other concepts have also been developed or adapted, such as decision rules [41], counterfactuals [42, 43], or contrastive explanations [43, 44] that are often (but not always) easier to interpret than feature attributions. For instance, in chemistry, counterfactuals represent close structural analogs with minimal chemical changes leading to opposing predictions, and contrastive explanations capture the smallest feature subsets responsible for different prediction outcomes [38, 43]. Figure 1 shows explanations obtained using different XAI approaches for an exemplary compound activity prediction.

**FIGURE 1:**
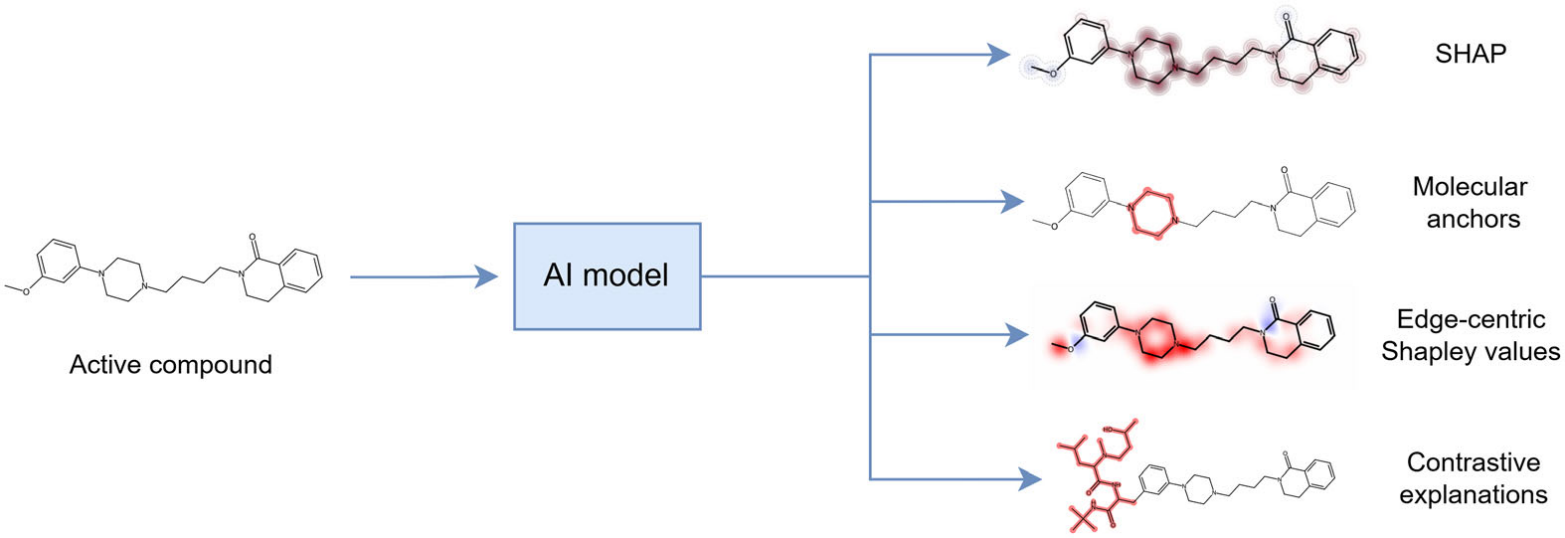
Alternative explanations for an exemplary compound activity prediction. Each XAI method applies different explanation criteria. Substructures identified by each approach to determine the correct prediction of activity of the test compound are highlighted. The different explanations identify corresponding molecular regions and provide complementary views on the model decision. SHAP is a widely used feature attribution approach. Molecular anchors are structural fragments that define the position of a compound in feature space and typically determine predictions. Edge‐centric Shapley values represent an adaptation of the SHAP formalism for graph neural networks to quantify the importance of edges for predictions. In medicinal chemistry, contrastive explanations identify molecular fragments that change prediction outcomes in a defined manner.

## 6 Scientific Foundation Models

The use of general‐purpose LLMs as a resource for scientific information, research planning, or generative modeling applications is often controversially viewed [45, 46]. LLMs are prone to hallucinations, that is, the generation of results that seem to be plausible but lack empirical support [47]. While techniques such as prompt engineering are generally applicable to reduce LLM hallucinations, specific scientific training or fine‐tuning of LLMs should, in principle, be a more comprehensive approach to control hallucinations in scientific applications. Therefore, models might be subjected to training with large amounts of domain‐specific scientific data and information to generate domain‐specific foundation models for different scientific disciplines that are less prone to scientific hallucinations. A variety of such models have already been generated for tasks in biology, chemistry, and drug discovery [48, 49, 50]. Domain‐specific scientific foundation models are not limited to LLMs but can have other DNN architectures. Notably, the term foundation model also applies to general‐purpose LLMs and implies wide generalization ability. However, most currently available scientific foundation models have been derived for specific tasks, whereas models with broad scientific domain coverage and multitasking ability within the domain are rare. Furthermore, as discussed below, the jury is out on whether scientific foundation models control hallucinations more effectively than frontier LLMs.

## 7 AI Agent Systems

Agentic AI can be understood as the development and application of AI systems to plan and execute multistep tasks with the aid of specific tools and present and evaluate the results in an increasingly autonomous manner. In the past 2–3 years, LLMs with or without specific scientific training have been used as a central component of AI agent systems for scientific applications [51, 52, 53]. For this purpose, it is also attempted to increase the reasoning capacity of LLMs through reinforcement or in‐context learning and other strategies [54, 55, 56]. While a generative model is derived for a given design task, a basic AI agent is a computational model for a specific function, typically executed with an associated tool. AI agent systems range from central models that sequentially deploy multiple tools to largely autonomous systems for addressing end‐to‐end research tasks using model‐internal or external agents with specific functions. The central component of an agent system is typically an LLM. Advanced AI agents communicate with the central LLM to, for example, formulate scientific hypotheses and design and execute sequences of tasks for hypothesis testing. Such AI agents often combine domain‐specific knowledge with decision‐making algorithms to minimize human intervention and move in the direction of autonomous systems [51, 52, 53]. At the leading edge currently are systems comprising a hierarchy of interacting AI agents trained to adopt different scientific roles [57] or individual LLM‐powered agents with multiple roles using different scientific foundation models [58]. Agent hierarchies consist of supervising (principal investigator) agents, others representing different scientists (domain experts), and critic or review agents that examine scientific outputs and provide a feedback loop for further refinements. Thus, these systems are designed to simulate interactions in a research team to address a scientific task [57]. Alternatively, based on LLM prompts, a single AI agent plans computational research, generates code, carries out the calculations, critically evaluates the results, and presents them in a manuscript format [58]. Such AI agent systems can address molecular design tasks with little human intervention [57]. At the next level, they could be interfaced with robotic platforms to carry out experimental work such as compound synthesis or expression of protein constructs [51, 52]. Figure 2 shows a possible workflow of an agentic AI system for scientific discovery.

**FIGURE 2:**
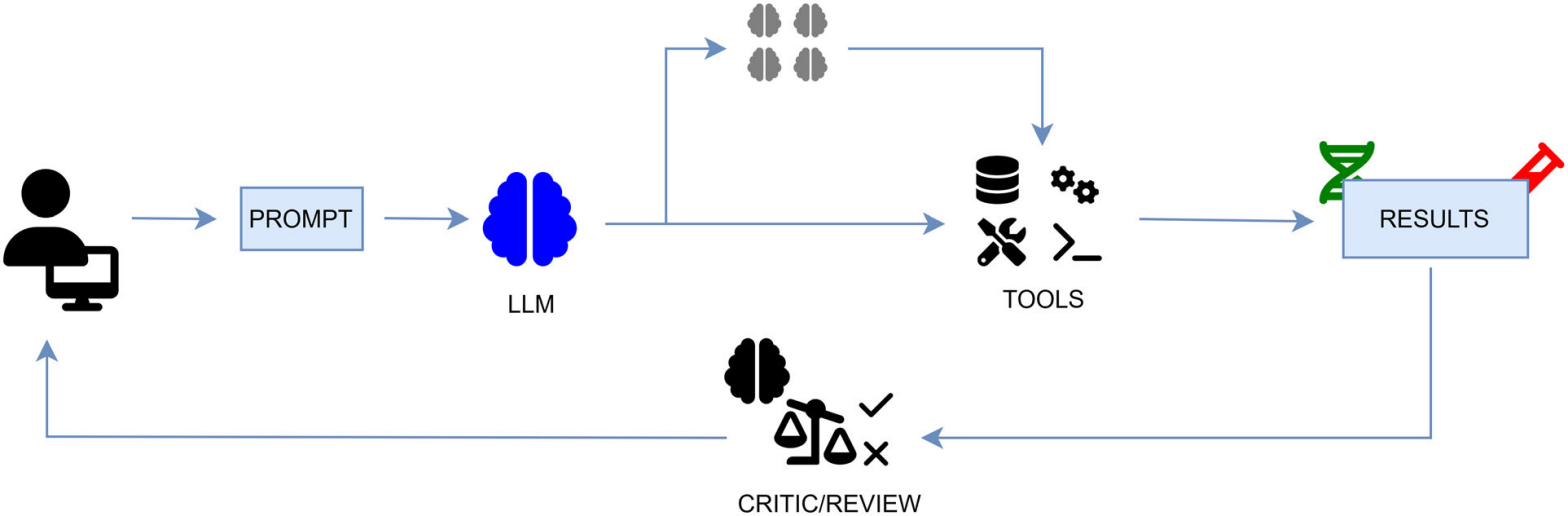
Exemplary workflow of an agentic AI system for scientific discovery. The user initiates the process with an LLM prompt. The central LLM can either interact with other LLMs or foundation models (agents) or directly invoke tools to address the task requested by a scientist. The results are analyzed by a critic/review agent providing scientific feedback before they are reported to the user.

## 8 Challenges

Compared to areas such as natural language processing or image analysis, drug discovery research is a data‐sparse domain, which does not play into the strength of deep learning. As a consequence, DNN‐based classification or regression models are not necessarily superior to standard machine learning models (such as support vector machines or decision tree‐based models), as shown by various studies [3]. Therefore, in drug discovery research and beyond, careful comparisons of complex DNN models with baseline methods are essential for the further development of machine learning methods. This also applies to the evaluation of deep docking methods including diffusion models [59]. Clearly, observing an increase in prediction accuracy of a few percent in a particular benchmark setting is insufficient to claim general superiority of a new and highly complex DNNs compared to simpler models.

However, going beyond property prediction, deep generative modeling has provided new opportunities for small molecule and protein design that significantly expand the capacity of conventional molecular design approaches [3, 60]. Notably, generative methods producing new molecules are difficult to benchmark. Reproducibility of known compounds (or analogs) with desired properties is often used as an evaluation criterion [16], which requires careful consideration. DNNs typically have high data memorization capacity [61], and training data memorization or extrapolation from similar training to test data has been observed for deep docking algorithms [59] or graph neural networks and transformers used for molecular activity predictions [62, 63]. Thus, for the evaluation of deep generative models, it is generally important to control any form of training‐to‐test data leakage [64].

For the current state‐of‐the‐art transformer and diffusion models, insights into their inner workings and explainability of predictions are still limited. Molecular diffusion models are just beginning to be explored from an XAI perspective [65]. For transformers, XAI studies have so far mostly focused on quantifying the importance of tokens or token relationships based on token perturbation, attention weights, or the determination of weight gradients [66, 67]. Attention weights of pairwise token combinations are visualized in heatmaps (token matrices). While these so‐called attention maps provide a global graphical view of token relationships, they are insufficient for explaining and interpreting predictions at the molecular level of detail. The current absence of readily interpretable XAI methods for molecular transformer models can be partly compensated for by careful control calculations based on specific input data modification that help to better understand transformer predictions [63]. Such control calculations and first XAI studies on molecular diffusion models have shown that neither transformer nor diffusion models are required to learn chemical or biological rules or concepts to arrive at apparently sound predictions [63, 65], contrary to often stated claims. Instead, the predictions usually entirely depend on detectable statistical data patterns and relationships, irrespective of the nature of the data.

The tendency of LLMs to produce scientific hallucinations severely affects their interactive use as research assistants or planners as well as LLM‐based AI agent systems. Although domain‐specific scientific foundation models should reduce hallucinations (see above), there currently are no detailed comparisons available for LLMs with and without specific scientific training. Much more work will be required to validate the use of LLM variants, especially taking into consideration recently introduced frontier LLMs with enhanced reasoning ability [68, 69] (a preferred choice for advanced AI agent systems).

## 9 Conclusion and Future Perspective

Herein, we summarize the development of AI models in early‐phase drug discovery over the past years and discuss associated challenges. Given that drug discovery is a data‐sparse domain compared to other fields where AI has a major impact, DNNs have not generally improved the performance of task‐specific machine learning. On the other hand, generative modeling adopting DNN architectures that excel in natural language processing or computer vision has provided new opportunities for drug discovery that are difficult or impossible to address with standard machine learning approaches. These include the design of synthetic reactions, compounds, and proteins. Transformer and diffusion models have become the preferred architectures for small molecule and protein design. Target structure‐based approaches represent a recent growth area, especially for equivariant DDPMs and the generation of new ligand–target complexes. Currently, much emphasis is put on new applications of transformer and diffusion models, whereas chemically and biologically intuitive XAI methods for these models are still lacking. However, such methods will be essential to better understand the learning characteristics of these models and their intrinsic limitations. In our view, there currently is a tendency to overinterpret the ability of these models to learn physical reality and scientific concepts from training data. This raises false expectations and ultimately hinders the integration of such models into interdisciplinary research environments.

A central challenge for drug discovery and other interdisciplinary research fields is the current pace at which novel developments occur. New generations of LLMs and efforts to further increase their reasoning abilities often cause rather uncritical use of these models for scientific applications. Moreover, the prototypic AI agent systems discussed herein were difficult to imagine just a few years ago. While many agent systems are still at their early stages and far away from a broader adoption, it is clear that these developments will continue at a fast pace (and likely further accelerate). Without doubt, AI agents will move in the direction of increasingly autonomous systems. This also applies to agents that are interfaced with robotic platforms to integrate molecular design and subsequent experimental work. For any of these AI agent architectures, rigorous validation efforts will be essential to evaluate their generalization ability and tightly control potential error sources and experimental hazards. Notably, given the complexity of AI agent architectures, conventional XAI methods such as feature attribution approaches, which are typically conceived for task‐specific predictive models, become much less relevant in this case. Instead, system‐level transparency is gaining importance, for example, through traceable tool use, data provenance, and uncertainty estimates. Furthermore, agent systems must meet the criteria of Trustworthy AI, that is, they must be reliable, transparent, safe, and secure and comply with ethical criteria for the conduct of research [70]. Here, safety and security requirements primarily apply to experimental activities and data (communication) issues, respectively. In drug discovery, the evaluation of AI agents should also concentrate on assessing reproducibility, task‐dependent performance compared to simple baselines, data leakage, error recovery, and the required degree of human supervision. There will be more future challenges. In high‐risk domains such as drug discovery, the advent of increasingly autonomous AI agents will require new regulatory/legal frameworks and clearly defined human control measures. Establishing such frameworks and measures is expected to take more time than will be required for the development of further advanced agentic AI and might complicate the wider adoption and distribution of these systems.

Last but not least, while our discussion has predominantly focused on molecular design, AI is expected to cover essentially the entire drug discovery and development pipeline in the foreseeable future. For instance, LLMs and more specialized foundation models are already used to aid in clinical trial planning and analysis as well as in the documentation of development efforts and the preparation of regulatory documents [71, 72]. Regardless of the applications along the drug discovery pipeline, the requirements for rigorous validation of AI models, as emphasized herein, remain the same as well as the need to demonstrate generalization ability beyond individual case studies.

## 10 Disclaimer

The opinions expressed in this publication are the view of the author(s) and do not necessarily reflect the opinions or views of ChemMedChem, the Publisher, ChemistryEurope, or the affiliated editors.

## Conflicts of Interest

The authors declare no conflicts of interest.

## Data Availability

Data sharing not applicable to this article as no datasets were generated or analysed during the current study.
